# Hair Dye

**Published:** 1870-11

**Authors:** 


### HAIR DYE.

Almost every article of this kind contains, in greater or less quantity, the most poisonous preparations of lead, which are absorbed into the scalp and lower face, become incorporated with the blood, and engender painful, incurable and disgusting diseases. It is said, in one of the French lunatic asylums, eight per cent. of the victims were users of hair dye. Gray hairs add dignity to man; those who are ashamed of age, and seek to conceal it in these ways, can scarcely claim the respect of their kind; and it would be wiser in them to be ashamed of their shallowness than of their age.

